# Evaluation of Free Amino Acid and Fatty Acid Concentrations in Simmental Calves Naturally Infected With *Mycoplasma bovis* Using a Metabolomics Approach

**DOI:** 10.1155/vmi/5135613

**Published:** 2026-06-27

**Authors:** Abdullah Gazi̇oğlu, Tuba Okutan, Ökkeş Yilmaz

**Affiliations:** ^1^ Food, Agriculture and Livestock Vocational School, Bingöl University, Bingöl, Turkey, bingol.edu.tr; ^2^ Faculty of Science, Department of Biology, Fırat University, Elazığ, Turkey, firat.edu.tr

**Keywords:** amino acid, calf, fatty acid, metabolomics, pneumonia

## Abstract

Amino acids and fatty acids are important metabolites that regulate immune responses and maintain body homeostasis. However, serum amino acid and fatty acid profiles in cattle naturally infected with *Mycoplasma bovis* (*M. bovis*) are unknown. This study aimed to evaluate changes in free fatty acid and amino acid concentrations in calves infected with *M. bovis*. In this study, the acute *M. bovis* group consisted of 20 Simmental calves of different sexes, aged 1–3 months, with respiratory disease symptoms. *M. bovis* was diagnosed by polymerase chain reaction (PCR). The control group consisted of 10 Simmental calves aged 1–3 months, which were deemed healthy following clinical examination. The concentrations of alanine, isoleucine, glutamic acid, lysine, histidine, and tyrosine in the *M. bovis* group were significantly lower than those in the control group. In contrast, valine and methionine levels in the *M. bovis* group were found to be significantly higher than those in the control group. The concentrations of lauric acid, pentadeconic acid, palmitic acid, DHA, and stearic acid were found to be significantly higher in the *M. bovis* group than in the control group. The levels of oleic acid and linoleic acid were found to be significantly lower in the *M. bovis* group than in the control group. In conclusion, it was determined that the free amino acid and fatty acid profiles in calves infected with *M. bovis* were significantly altered and that these metabolites play an important role in the pathogenesis of *M. bovis* infection in animals. In addition, the low concentrations of some amino acids observed in this study suggest they may have therapeutic potential in the future.

## 1. Introduction

The respiratory system is a complex structure that allows living organisms to take in oxygen, distribute it throughout the body, and remove carbon dioxide [1]. This structure facilitates humidification, heating, and particle removal from inhaled air [2]. While the respiratory system performs the processes mentioned above, it also plays an important role in regulating acid–base balance and blood pressure to maintain homeostasis [3].

The reasons why respiratory diseases are more common in calves than in other species include the highly lobulated structure of the lungs, low gas‐exchange capacity, and high sensitivity to environmental temperature changes [4]. Bovine respiratory disease (BRD) complex is a multifactorial condition resulting from the interaction between management‐related factors (such as care‐feeding, environmental conditions, and stress) and infectious agents (including bacterial and viral pathogens) [1, 2]. One of the commonly observed agents in cattle is *Mycoplasma bovis* (*M. bovis*).

Animals infected with *M. bovis* commonly present with severe necrosuppurative bronchopneumonia, fibrinonecrotizing pneumonia, or mild catarrhal bronchointerstitial pneumonia when exposed to low infectious doses [5]. Lung necropsies of calves that died following natural infection with *M. bovis* have been reported to show exudative bronchopneumonia and extensive foci of coagulation necrosis surrounded by neutrophils, monocytes, and lymphocytes [5, 6].

The cost of respiratory diseases was reported at $42.15 per calf in the study by Dubrovsky et al. [7]. In addition, Wang et al. [8] estimated that the total cost of pneumonia in calves between 2011 and 2015 reached $165 million.

The omics approach is widely preferred because it enables the development of new nutritional, genetic, diagnostic, and treatment strategies [9–11]. Metabolomic analysis, one of the omics technologies, is a powerful technique that allows the analysis of all metabolites present in biological fluids (milk, urine, and blood) and tissues throughout the body. This approach facilitates understanding of how metabolites respond to various disease conditions [10, 12]. Since amino acids (AAs) and fatty acids are the basic building blocks of proteins and fats, they play essential roles in intermediary metabolism and act as precursors to numerous biomolecules [9].

The main reason free AAs and fatty acids change in many disease states is their active role in systemic metabolic pathways and organ functions [10–12]. This study aimed to determine the free fatty acid and AA profiles in calves with *M. bovis* and to evaluate the changes in their concentrations associated with *M. bovis* infection.

## 2. Materials and Methods

Before starting the study, the necessary ethical permissions were obtained from the Bingöl University Experimental Animals Local Ethics Committee (Meeting Number: 2024/02, Decision Number: 02/01).

### 2.1. Animals

This study was a cross‐sectional study. Blood samples were collected from the animals only once. This study included 20 Simmental calves aged 1–3 months with respiratory disease. In the study, 12 of the 20 infected cattle were male, and 8 were female, while the control group of 10 calves consisted of 6 males and 4 females. The average age of calves in the *M. bovis* group was 62 ± 8 days, while the average age of calves in the control group was 58 ± 12 days. The calves used in this study were obtained from a farm in Bingöl Province. The control group was also obtained from the same farm. The information and records obtained from the animal owner indicated that the calves were not vaccinated against respiratory diseases. The animals were housed on a farm under semi‐open conditions.

The control group consisted of 10 healthy calves aged 1–3 months, determined by clinical examination. In the evaluation of calves, body temperature, heart rate, and respiratory rate, as well as the presence or absence of diarrhea, cough, nasal discharge, and ocular discharge, were assessed. Behavioral changes, sucking reflex, activity, and appetite were also assessed, and thoracic auscultation was performed. The cattle in the study were considered healthy according to clinical examination parameters (heart rate [beats per minute], respiratory rate [breaths per minute], and body temperature [°C]) and laboratory analyses (hematological, biochemical, and blood gas analyses). The study excluded animals that had received antibiotics within the previous week or had congenital or acquired conditions other than pneumonia, based on the animal owner’s anamnesis.

### 2.2. Collection of Blood Samples

To determine the free AA (alanine, glycine, valine, leucine, isoleucine, proline, methionine, serine, cysteine, tyrosine, and histidine) and fatty acid profile (lauric acid, pentadeconic acid, palmitic acid, docosahexaenoic acid [DHA], stearic acid, oleic acid, linoleic acid, margaric acid, heptadecenoic acid, myristic acid, pentadecylic acid, eicosatrienoic acid, and arachidonic acid) of the *M. bovis* and control group calves, 8 mL of blood was collected from the jugular vein into tubes without anticoagulant (BD Vacutainer®, Plymouth, UK) using standard procedures. Blood samples were left at room temperature to clot and then centrifuged at 3000 rpm for 5 min (Hermle Z 36 HK®, Germany) to obtain serum. To analyze serum AA concentration, gas chromatography with a flame ionization detector (GC‐FID) was used for fatty acid analysis. For hematological analyses, blood was collected in 3‐mL anticoagulant tubes containing K_3_EDTA (BD Vacutainer, Plymouth, UK). Total leukocyte (WBC) counts in complete blood count were performed on an automatic hematology analyzer (BeneSphera H‐31, USA).

### 2.3. *M. bovis* Diagnosis

In this study, nasal swab samples were used to determine *M. bovis*. Nasal swab samples were analyzed by polymerase chain reaction (PCR). The phenol–chloroform method was used in DNA isolation for molecular identification of samples. The lysed or homogenized sample was transferred into a microcentrifuge tube, and an equal volume of phenol–chloroform (1:1) was added. The phenol used in the extraction was equilibrated to pH 8.0. Isoamyl alcohol was added to chloroform (24:1) as an antifoaming agent. The mixture was vortexed thoroughly. The tubes were centrifuged at room temperature for 1 min at high speed. Two phases were formed, with the upper aqueous phase containing DNA and the lower hydrophobic phase containing proteins. The aqueous phase was transferred to a new tube, and an equal volume of chloroform–isoamyl alcohol was added to remove residual phenol. The mixture was vortexed and centrifuged again, and the aqueous phase was transferred to a new tube. An equal volume of ice‐cold 100% ethanol was added for DNA precipitation. The sample was centrifuged to obtain a pellet, and the supernatant was discarded. The pellet was washed with 1 mL of 70% ethanol and centrifuged again at high speed for 1 min. The supernatant was removed, and the pellet was air‐dried and resuspended in molecular biology‐grade water [13].

In this study, primers were designed using Primer‐BLAST (NCBI), which enables the generation of target‐specific primers from sequence input.

Base sequence of *M. bovis* primers (498 bp).

F 5′‐ ATTGAATCAGGTCAGCCAAA ‐ 3′

R 5′‐ TCCATCAGAAACATCAAGCA ‐ 3′

PCR cycles were performed as follows: enzyme activation at 95°C for 2–5 min, denaturation at 95°C for 10 s, binding at 60°C for 60 s, extension at 72°C for 60 s, and a final extension at 72°C for 2 min.

### 2.4. AA Analysis

After washing the serum samples with 0.9% NaCl, the wet weights were determined, and the samples were homogenized in 10 mL of 50 mM Tris–20 mM EDTA (pH 7.4) buffer. The samples were then centrifuged at 4°C for 9 min at 9000 rpm. After centrifugation, 50 μL of the supernatant was collected for total protein determination. Derivatization of AAs with N‐(t‐butyldimethylsilyl)‐N‐methyltrifluoroacetamide (MTBSTFA) produces simultaneous silylation of amino and carboxyl groups in a single step according to a previously described modification procedure [14].

The protein fraction of the serum sample was precipitated with 10% TCA, and the solvent was evaporated to dryness under a nitrogen stream. Then, 60 μL of acetonitrile and 60 μL of MTBSTFA were added, and the test tube was sealed with a Teflon cap. The mixture was incubated at 70°C for 20 min to derivatize the AAs and then cooled. Subsequently, 600 μL of chloroform was added, and the mixture was homogenized; the resulting mixture was analyzed by GC‐FID for AA derivatives. AA standards were prepared under the same conditions.

For the analysis of AA derivatives, a Shimadzu gas chromatograph (2010 Plus), adapted for glass‐capillary operations and equipped with a FID, was employed. The AA derivatives were separated on a 20 m Supelco SLB‐5 ms capillary column (Supelco, Sigma; 0.25 mm ID and 0.25 μm film thickness). Helium was used as the carrier gas at a flow rate of 45 cm/sec. The temperature program was as follows: initial temperature of 120°C, increased to 150°C at 120°C/min (held for 5 min), then increased to 240°C at 7°C/min, and finally increased to 285°C at 20°C/min (held for 18 min). The injector and detector temperatures were maintained at 240°C and 300°C, respectively.

The identification of AA derivatives was performed by comparing their FID chromatograms and retention times with those of authentic reference standards. The percentage composition of AA derivatives was calculated using the LabSolutions LC/GC 5.91 software (Shimadzu, Kyoto, Japan).

### 2.5. Analysis of Fatty Acid Levels by Gas Chromatography

Total lipid contents of serum samples were extracted after homogenization in a 3:2 (v/v) hexane–isopropanol mixture according to the procedure described by Hara and Radin. All solvents contained 0.01% butylated hydroxytoluene as an antioxidant. Fatty acid methyl esters were prepared from total lipid by acid‐catalyzed transmethylation at 55°C for 12 h according to the method of Christie [15].

The fatty acid methyl esters formed in the tubes were extracted with 5 mL of hexane; the hexane phase was collected from the upper layer with a pipette, treated with 5 mL of 2% KHCO_3_, and left for 24 h. The solvent was evaporated in an oven at 37°C, and the remaining residue was dissolved in 1 mL of chloroform and analyzed by gas chromatography [15].

Fatty acid analyses were performed using gas chromatography with an FID detector. An Rtx® 2330 GC column (30 m, 0.25 mm ID, 0.25 μm df; Sigma, USA) was used. Before the analysis of sample fatty acid methyl esters, standard mixtures of fatty acid methyl esters were injected, and the methyl esters of fatty acids were identified by comparison with authentic external standard mixtures analyzed under the same conditions.

The percentage composition of fatty acid methyl esters was calculated using the LabSolutions LC/GC 5.91 operating program (Shimadzu, Kyoto, Japan). Supelco 37 Component FAME Mix was used as the FAME standard mixture. During the analysis, the column temperature was set to 90°C–218°C, the injection temperature to 245°C, and the detector temperature to 285°C. The column temperature program was adjusted from 90°C to 218°C. Helium gas was used as the carrier gas.

The percentage composition of fatty acid methyl esters was calculated using LabSolutions LC/GC 5.91 software (Shimadzu, Kyoto, Japan).

### 2.6. Statistical Analysis

Statistical analysis of the collected data was conducted using SPSS 26 (IBM SPSS Statistics for Windows, Version 26.0, Armonk, NY: IBM Corp.) and GraphPad Prism (Prism 9 for Windows, Version 9). In this study, the G∗Power statistical analysis tool was used. The sample size was determined to be 30 subjects based on an effect size of 0.9, a type I error rate of 0.05, and a statistical power of 80%. Data were presented as mean ± standard deviation (SD).

The Shapiro–Wilk test was used to evaluate whether the data followed a normal distribution. To determine differences between the *M. bovis* and control groups, the Mann–Whitney *U* test was used for non‐normally distributed data, and the independent‐samples *t*‐test was used for normally distributed data. In this study, the relationship between leukocytes and AAs and fatty acids was determined using Spearman’s rank correlation analysis. The significance level was set at *p* < 0.05.

## 3. Results

**FIGURE 1:**
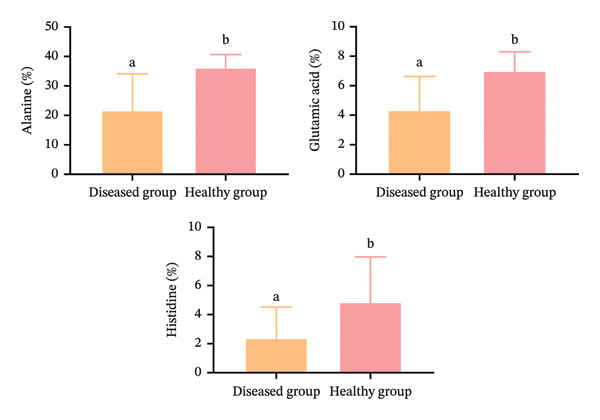
Free amino acid values of the diseased and healthy groups.

Table 1 and Figure 1 present the clinical examination findings of the *M. bovis* and control groups. No statistically significant difference was found in heart rate (*p* > 0.334) and body temperature (*p* > 0.417) between the *M. bovis* and control groups. Respiratory rate (*p* < 0.046) and leukocyte count (*p* < 0.001) were found to be significantly higher in the *M. bovis* group than in the control group.

**TABLE 1:** Means of clinical findings in diseased and healthy calves and the statistical significance levels between groups.

Variables	Diseased	Healthy	*p* value
Heart frequency	105.05 ± 20.15	98.30 ± 11.05	0.334
Respiratory frequency	43.30 ± 19.88^a^	33.60 ± 3.50^b^	0.046
Body temperature	38.85 ± 1.09	38.55 ± 0.47	0.417
Leukocyte	20.36 ± 11.92^a^	7.30 ± 2.01^b^	0.001

*Note:* Different superscript letters (a, b) within the same row indicate statistically significant differences between groups (*p* < 0.05). Data are presented as mean ± standard deviation (SD).

**FIGURE 2:**
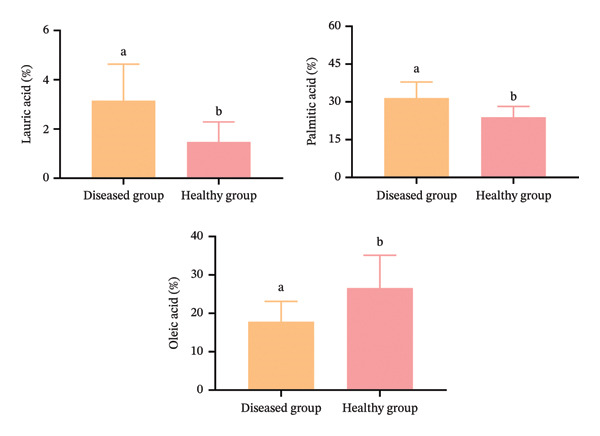
Free fatty acid values of the diseased and healthy groups.

Table 2 and Figure 2 display the free AA values of the *M. bovis* and control groups. Alanine (*p* < 0.001), isoleucine (*p* < 0.003), glutamic acid (*p* < 0.002), lysine (*p* < 0.006), histidine (*p* < 0.018), and tyrosine (*p* < 0.001) concentrations in the *M. bovis* group were significantly lower than those in the control group. In contrast, valine (*p* < 0.003) and methionine (*p* < 0.002) levels were found to be significantly higher in the *M. bovis* group than in the control group. No statistically significant difference was found between the *M. bovis* and control groups in terms of proline (*p* > 0.397), serine (*p* > 0.914), threonine (*p* > 0.210), phenylalanine (*p* > 0.086), aspartic acid (*p* > 0.564), cysteine (*p* > 0.930), tryptophan (*p* > 0.187), glycine (*p* > 0.098), and leucine (*p* > 0.365) concentrations.

**TABLE 2:** Mean free amino acid values in diseased and healthy calves and the statistical significance levels between groups.

Amino acids	Groups			
EAA**^†^**	Unit	Diseased	Healthy	*p* value
Histidine	%	2.39 ± 2.18^a^	4.48 ± 3.11^b^	0.018
Isoleucine	%	3.41 ± 1.90^a^	4.97 ± 0.87^b^	0.003
Leucine	%	5.23 ± 2.91	6.13 ± 1.69	0.365
Methionine	%	3.29 ± 8.54^a^	2.57 ± 0.68^b^	0.002
Phenylalanine	%	8.30 ± 7.02	9.84 ± 1.87	0.086
Threonine	%	1.35 ± 1.08	0.84 ± 0.33	0.210
Valine	%	29.90 ± 26.34^a^	5.61 ± 4.20^b^	0.003
NEAA^‡^				
Alanine	%	21.74 ± 12.66^a^	35.38 ± 4.07^b^	0.001
Aspartic acid	%	2.01 ± 1.40	1.77 ± 0.74	0.564
Cysteine	%	1.84 ± 2.16	1.08 ± 0.50	0.930
Glutamic acid	%	4.35 ± 2.33^a^	6.99 ± 1.43^b^	0.002
Glycine	%	4.90 ± 2.49	6.67 ± 2.44	0.098
Lysine	%	1.12 ± 1.33a	2.98 ± 2.76	0.006
Proline	%	4.38 ± 5.33	3.72 ± 1.35	0.397
Serin	%	1.88 ± 1.06	2.21 ± 0.77	0.914
Tyrosine	%	1.07 ± 0.66^a^	2.16 ± 0.43^b^	0.001
Tryptophan	%	0.97 ± 0.92	1.02 ± 0.32	0.187

*Note:* Different superscript letters (a, b) within the same row indicate statistically significant differences between groups (*p* < 0.05). Data are presented as mean ± standard deviation (SD).

^†^Essential amino acids.

^‡^nonessential amino acids.

Table 3 displays the free fatty acid values of the *M. bovis* and control groups. The concentrations of lauric acid (*p* < 0.001), pentadeconic acid (*p* < 0.009), palmitic acid (*p* < 0.001), DHA (*p* < 0.006), and stearic acid (*p* < 0.009) in the *M. bovis* group were found to be significantly higher than those in the control group. The levels of oleic acid (*p* < 0.002) and linoleic acid (*p* < 0.006) in the *M. bovis* group were found to be significantly lower than those in the control group. No statistically significant difference was found between the *M. bovis* and control groups in terms of margaric acid (*p* > 0.202), heptadecenoic acid (*p* > 0.654), myristic acid (*p* > 0.333), pentadecylic acid (*p* > 0.671), eicosapentaenoic acid (*p* > 0.111), and arachidonic acid concentrations. No statistically significant relationship was found between leukocytes and AAs or fatty acids.

**TABLE 3:** Mean free fatty acid values in diseased and healthy calves and the statistical significance levels between groups.

Fatty acids	Unit	Diseased	Healthy	*p* value
Arachidonic acid	%	2.63 ± 3.14	2.30 ± 3.15	0.187
DHA	%	1.54 ± 1.35^a^	0.15 ± 0.18^b^	0.006
Eicosatrienoic acid	%	0.99 ± 0.39	0.75 ± 0.42	0.111
Heptadecenoic acid	%	0.34 ± 0.12	0.36 ± 0.21	0.654
Lauric acid	%	3.16 ± 1.48^a^	1.53 ± 0.84^b^	0.001
Linoleic acid	%	8.06 ± 5.83^a^	15.90 ± 9.84^b^	0.006
Margaric acid	%	1.24 ± 0.44	1.16 ± 0.51	0.202
Myristic acid	%	1.43 ± 0.73	1.12 ± 0.54	0.333
Oleic acid	%	17.81 ± 5.29^a^	26.01 ± 8.81^b^	0.002
Palmitic acid	%	31.51 ± 6.34^a^	23.70 ± 4.4^b^	0.001
Pentadecanoic acid	%	0.58 ± 0.30^a^	0.28 ± 0.20^b^	0.009
Pentadecylic acid	%	0.65 ± 1.19	0.71 ± 0.3	0.671
Palmitoleic acid	%	1.10 ± 0.40	1.31 ± 0.62	0.189
Stearic acid	%	26.75 ± 5.01^a^	20.71 ± 7.75^b^	0.009

*Note:* Different superscript letters (a, b) within the same row indicate statistically significant differences between groups (*p* < 0.05). Data are presented as mean ± standard deviation (SD).

## 4. Discussion

AAs and fatty acids, which are precursors of many biomolecules in the body, play a role in the execution of essential physiological functions. The main reason for changes in free AAs and fatty acids across many disease conditions is their active roles in systemic organs and pathways [9, 12]. Therefore, this study aimed to evaluate the changes in free AA and fatty acid levels in calves with *M. bovis*.

There are significant discrepancies in the literature regarding leukocyte counts in the respiratory system and various other diseases [16–19]. In a study by Dudek et al. [16] that experimentally induced *M. bovis* infection in calves, leukocyte counts did not differ significantly between the control and experimental groups. In contrast, the study by Ider and Maden [17] reported that leukocyte counts were significantly higher in calves with fibrinous and caseonecrotic pneumonia. In this study, as in the findings of Ider and Maden [17], the leukocyte count in the *M. bovis* group was significantly higher than that in the control group. It is thought that this difference between the studies may be related to differences in measurement methods, the acute or chronic stage of the infection, and variations in etiological factors.

In recent years, the development of analytical methods has enabled the comprehensive identification of a wide range of metabolites across different samples, such as serum, plasma, and tissues [20, 21]. The metabolomics approach enables the analysis of metabolites in complex diseases and facilitates the identification of those altered during disease processes [21]. One of these metabolites, AAs, acts as a mediator of host–pathogen interactions, and their levels significantly affect the course of pathogenic infection [22].

Glucogenic AAs, such as alanine, glycine, arginine, serine, threonine, and tryptophan, are metabolized to produce pyruvic acid. These AAs contribute to the formation of intermediates, such as oxaloacetic acid, fumaric acid, *α*‐ketoglutarate, pyruvic acid, and succinyl‐CoA, which in turn participate in energy production pathways [21, 22]. It has been reported that the concentration of alanine is significantly lower in people naturally infected with bacterial pneumonia [21]. In parallel with this study, Pouw et al. [23] and Yoneda et al. [24] reported that alanine levels are significantly low in people with chronic obstructive pulmonary disease. In a study of naturally infected calves with pneumonia, the alanine concentration was significantly lower than in the control group. It was stated that the probable reason for this decrease in alanine concentration was anorexia and inflammation, which altered gluconeogenesis and muscle anabolic or catabolic processes [6]. In this study, the alanine concentration in the *M. bovis* group was significantly lower than that in the control group, as supported by previous studies.

Studies on the pathophysiology of branched‐chain AAs, such as leucine, isoleucine, and valine, have reported that they are essential AAs that play an important role in energy metabolism, hepatic disorders, intestinal health, and immune system regulation in the body [6, 22, 24]. These AAs are broken down in skeletal muscles and contribute to glycogen synthesis and storage in energy metabolism [20, 22]. In a study of patients with chronic obstructive pulmonary disease, valine and leucine concentrations were significantly reduced [24]. Similarly, in another study conducted by Tsukano et al. [6], it was reported that the concentrations of branched‐chain AAs valine and leucine were significantly decreased in calves with Mycoplasma bronchopneumonia. It was also stated that decreased serum valine and leucine levels in hypermetabolic disorders may be associated with pulmonary dysfunction and muscle weakness. Lawrence et al. [25] and Imbery et al. [26] reported that valine and leucine concentrations were significantly decreased in dogs with various hepatic diseases. Since branched‐chain AAs are not metabolized in the liver, their concentrations are reported to decrease in liver disease [6].

In this study, it was determined that isoleucine concentration was significantly decreased in the calves in the patient group, in accordance with the literature reports. In addition, although the leucine level in the patient group was numerically lower than in healthy animals, no statistically significant difference was observed between the groups.

Histidine has anti‐inflammatory and antioxidant properties, as it suppresses the expression of pro‐inflammatory cytokines and removes reactive oxygen species during acute inflammation [27]. Ikeda [21] reported a significant decrease in histidine concentration in patients with bacterial pneumonia. In a study investigating AA concentrations in patients with community‐acquired pneumonia and chronic obstructive pulmonary disease, histidine levels decreased significantly in both groups, and histidine showed very strong diagnostic performance for community‐acquired pneumonia [27]. Similarly, in this study, consistent with previous literature, the histidine concentration in the *M. bovis* group was significantly lower than in the control group.

It has been reported that a decrease in histamine concentration is associated with histamine catabolism by mast cells and by immune and nonimmune cells [27]. This finding may also be associated with the inflammatory response observed in the calves in this study and the utilization of histamine at different stages of the disease process.

The AAs, arginine, glutamine, proline, and histidine are metabolized to glutamic acid, an important nutrient for immune cells [21]. In addition, this AA is an important energy source for leukocytes and fibroblasts and is significantly metabolized by both the kidneys and the intestines [9]. In a study of patients with chronic obstructive pulmonary disease, glutamic acid concentrations were significantly lower in both blood and muscle tissue [23]. Morrison et al. [28] and Schols et al. [29] reported significantly lower glutamic acid levels in patients with chronic obstructive pulmonary disease.

This study is also consistent with previous literature, which found that the glutamic acid concentration in the *M. bovis* group was significantly lower than that in the control group. The possible reason for this decrease may be related to the relationship between the hypermetabolic state and glutamic acid consumption during the development of systemic inflammation [9].

Methionine plays an important role in regulating many biological processes, including glutathione formation, polyamine synthesis, and methylation. It also helps eliminate oxidized metabolites, such as hydrogen peroxide, hydroxyl radicals, and chloramines, in many physiological processes [30]. Arshad et al. [27] reported that methionine concentration was significantly increased in patients with chronic obstructive pulmonary disease and showed very good performance in distinguishing community‐acquired pneumonia from chronic obstructive pulmonary disease patients.

In contrast, a study conducted by Chan et al. [31] reported that plasma methionine concentrations were significantly lower in dogs with pancreatitis, sepsis, and trauma compared to the control group. Similarly, in a study of patients with community‐acquired pneumonia, methionine concentration was significantly lower [21]. In a study conducted by Malmezat et al. [32] on rats experimentally induced with sepsis, it was reported that the concentration of methionine was significantly increased.

In this study, the methionine concentration in the *M. bovis* group was significantly higher, consistent with Arshad et al. [27]. The differences in results between this study and those of Malmezat et al. [32], Ikeda [21], and Chan et al. [31] may be due to differences in disease type, analytical methods, species, and preanalytical conditions.

Fatty acids play an important role in many processes, including AA production, cellular communication, gene expression, inflammation, and lipoprotein metabolism [33]. Lipids constitute 90% of the surfactant substance in the lung, and changes in the lipid profile occur in many respiratory diseases associated with pneumonia [33, 34]. Many studies have shown that lipid metabolism is significantly impaired in sepsis [35], bacteremia [36], and viral diseases [34] and that lipids are important mediators of inflammation.

In a study of 21 women with preeclampsia, the concentrations of total free fatty acids, palmitic acid, palmitoleic acid, stearic acid, oleic acid, and linoleic acid were significantly increased [37]. In a study conducted by Feng et al. [38] on 240 patients with nonalcoholic fatty liver disease, it was determined that the palmitic acid concentration of the obese group was significantly higher than that of healthy and lean individuals, and this fatty acid also showed a significant positive correlation with systolic blood pressure, diastolic blood pressure, and HOMA‐IR.

Similarly, in a study of patients with community‐acquired pneumonia, palmitic and oleic acid concentrations tended to increase, while linoleic and arachidonic acid levels decreased [33]. In this study, in accordance with previous literature reports [34, 36–38], it was determined that the concentrations of lauric acid, pentadecanoic acid, stearic acid, and DHA in the *M. bovis* group were significantly higher and that the fatty acid composition in calves with pneumonia was significantly altered.

It is thought that the change in the fatty acid profile in this study may be related to the development of inflammation and the formation of a negative energy balance in the animals.

A study on pneumonia showed significant changes in the AA profile but no significant changes in leukocyte counts [21]. Since there are no direct studies evaluating the relationship between fatty acids and leukocytes in pneumonia, no further assessment could be made. In this study, no significant relationship was found between leukocytes and AAs or fatty acids. The possible reason for the lack of a statistically significant association between leukocytes and AAs or fatty acids in this study may be related to the fact that the inflammatory response and systemic AA remodeling in cattle with pneumonia involve distinct mechanisms and pathways [21, 27, 39].

This study has some limitations. First, blood was collected only once in this study, which represents an important limitation. Second, pneumonia status was evaluated solely on clinical findings, and lung ultrasonography or radiography was not performed, which significantly limits lung evaluation. In cattle with *M. bovis* pneumonia, it would be more appropriate to perform AA and fatty acid analyses at multiple time points rather than at a single time point. The study would benefit from a larger sample and a wider range of pneumonia types.

## 5. Conclusions

We concluded that calves with *M. bovis* had significantly altered free AA and fatty acid profiles, which may play an important role in the pathogenesis of *M. bovis*. In the future, it is necessary to investigate the therapeutic and prognostic properties of amino and fatty acids in larger populations.

## Author Contributions

Abdullah Gazioğlu collected the study materials, developed the hypothesis, wrote the article, and performed the statistical analysis. Tuba Okutan and Ökkeş Yılmaz conducted the laboratory analyses of the study.

## Funding

This research did not receive any specific grant from funding agencies in the public, commercial, or not‐for‐profit sectors.

## Disclosure

All authors read and approved the final version of the manuscript.

## Conflicts of Interest

The authors declare no conflicts of interest.

## Data Availability

The data that support the findings of this study are available on request from the corresponding author. The data are not publicly available due to privacy or ethical restrictions.
